# Novel Acylated Naringin Enhances Propionate Release and Stimulates the Growth of Flavanone-Metabolizing Bacteria in an In Vitro Batch Fermentation Model

**DOI:** 10.3390/life15060967

**Published:** 2025-06-17

**Authors:** Blanca Elizabeth Ruiz-Álvarez, José Daniel Padilla-de la Rosa, Marisela González Avila, Georgina Sandoval, Yves Desjardins

**Affiliations:** 1 Institute sur la Nutrition et les Aliments Fonctionnels (INAF) de l’Université Laval, Faculté des Sciences de l’Agriculture et de l’Alimentation, Université Laval, Québec, QC G1V 0A6, Canada; blanca-elizabeth.ruiz-alvarez.1@ulaval.ca; 2 Centre de Recherche Nutrition, Santé et Société (NUTRISS), INAF Université Laval, Québec, QC G1V 0A6, Canada; 3 Centro de Investigación y Asistencia en Tecnología y Diseño del Estado de Jalisco A. C. (CIATEJ), Guadalajara 44270, Jalisco, Mexico

**Keywords:** SCFAs, gut microbiota, naringin, acylated SCFA molecules

## Abstract

The increasing prevalence of non-communicable diseases (NCDs) is strongly associated with gut microbiota (GM) imbalances and reduced short-chain fatty acid (SCFA) production, primarily driven by poor diet and microbial dysbiosis. Since SCFAs are crucial for gut health, immune regulation, and inflammation control, restoring their levels is a key therapeutic target. SCFA-acylated naringin derivatives offer a novel approach by enhancing SCFA delivery and modulating GM composition. In this study, we investigated the effects of naringin acetate and naringin propionate on SCFA production using a 24 h short-term in vitro batch fecal fermentation model with microbiota from two donors. Naringin propionate and naringin plus free propionate significantly increased propionate levels by 0.74 mM and 0.75 mM, respectively (*p* < 0.0001), while naringin acetate induced a smaller increase of 0.26 mM. Donor-specific reflected differences in microbial communities, yet SCFA enhancement was observed across samples. Additionally, naringin treatments stimulated the growth of beneficial polyphenol-metabolizing bacteria, including *Bacteroides*, *Streptococcus*, and *Eubacterium siraeum*. The strong effect of naringin propionate suggests a sustained SCFA release mediated by microbial enzymes. These preliminary results highlight the potential of SCFA-acylated flavonoids as functional dietary components to increase SCFA bioavailability and support gut health, particularly from citrus-derived co-products.

## 1. Introduction

Over the past two decades, the global prevalence of non-communicable diseases (NCDs) has risen sharply, driven by sedentary lifestyles, excessive calorie intake, poor dietary quality, and chronic stress [1]. These factors not only contribute to the onset of chronic diseases but also disrupt gut microbiota (GM) composition, causing an imbalance in microbial homeostasis and affecting the production of key metabolites, particularly short-chain fatty acids (SCFAs). The production of SCFAs is closely linked to the abundance of specific microbial populations [2,3]. Among SCFAs, propionate holds particular importance for health by supporting immune function, lowering serum cholesterol levels, enhancing satiety through the regulation of gut-derived hormones, and exerting anti-inflammatory effects in both the gut and the brain, potentially reducing neuroinflammation [4,5]. Conversely, butyrate has been extensively studied for its anti-inflammatory and anti-carcinogenic properties, as well as its essential role in preserving gut integrity by supporting epithelial barrier function, modulating immune responses, and maintaining mucosal homeostasis [6,7]. SCFA production is significantly impaired by insufficient dietary fiber intake, which deprives GM of fermentable substrates and is further diminished by dysbiosis resulting from factors such as antibiotic use or conditions like inflammatory bowel disease (IBD) [8,9]. Restoring adequate levels of SCFAs presents challenges; for instance, propionate synthesis relies on distinct microbial pathways, including the acrylate, succinate, and propanediol pathways, which are independent of the pathways involved in the production of other SCFAs, thereby limiting its production via cross-feeding. In contrast, acetate and butyrate production is metabolically interconnected: acetate serves as a key precursor for butyrate synthesis in certain microbial species, while butyrate-producing bacteria can utilize accumulated acetate for their metabolic processes [10].

Manipulating SCFA production in the gut through various interventions is challenging, as it relies on the availability of specific fermentable substrates, primarily dietary carbohydrates, and the presence of specialized bacterial taxa capable of synthesizing these metabolites. A promising targeted strategy involves the use of SCFA-conjugated molecules, which can directly deliver these metabolites to the intestinal environment while simultaneously modulating GM composition and offering additional bioactive benefits. The successful application of inulin-propionate ester, which has demonstrated potential for enhancing SCFAs release and modulating GM by reducing Clostridia and increasing Actinobacteria, underscores the promise of functional compounds in shaping microbial composition and metabolite production [11]. Interestingly, naringin, a glycosylated flavanone abundant in citrus fruits, possesses a unique chemical structure with multiple hydroxyl groups, making it particularly amenable to acylation with SCFAs. Leveraging these structural features, our research group has synthesized novel SCFA-acylated naringin derivatives from citrus co-products designed to enhance SCFA delivery, modulate GM composition, and provide additional bioactive properties [12].

We specifically aimed to evaluate the ability of naringin propionate to increase propionate concentrations in the colon compared to a control and to assess whether it could act as an exogenous source of propionate influencing GM homeostasis. In parallel, we investigated whether naringin acetate could enhance acetate concentrations and subsequently promote butyrate production via cross-feeding through the Butyryl-CoA: acetate CoA-transferase pathway. Additionally, we examined the potential of both SCFA-acylated derivatives to positively influence GM diversity and improve the bioaccessibility and bioactivity of naringin by promoting its microbial deglycosylation to naringenin. We further assessed whether these compounds could support the growth of propionate- and butyrate-producing bacteria while reducing the abundance of opportunistic taxa. To test these hypotheses, we conducted a two-stage in vitro batch fecal fermentation, simulating gastrointestinal conditions to evaluate their effects on GM composition and metabolic activity.

## 2. Materials and Methods

### 2.1. SCFA-Acylated Naringin Derivatives

**Figure 1:**
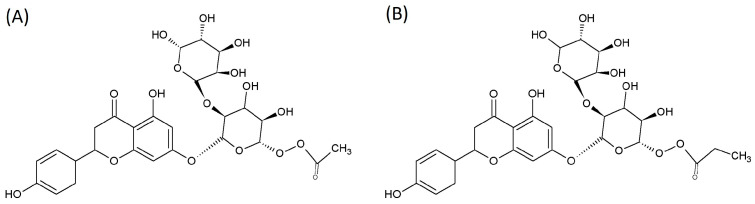
Chemical structure of acylated naringin compounds. (**A**): Naringin acetate; (**B**): naringin propionate.

Enzymatically acylated naringin derivatives—vinyl acetate naringin and vinyl propionate naringin—were obtained from Gutiérrez-Navarro et al. [12], shown in Table 1 and Figure 1. Vinyl propionate, vinyl acetate, and 98% pure naringin were sourced from Sigma-Aldrich (Oakville, Canada). Reagents used for the pre-digestion stage, conducted in the Simulator of the Human Intestinal Microbial Ecosystem (SHIME^®^), are listed in the Supplementary Materials.

### 2.2. Fecal Fermentation

Two participants with contrasting GM were recruited to donate fecal samples for a batch fermentation experiment. Both participants provided informed consent, following the guidelines of the Ethics Committee for Research Involving Human Beings at Université Laval (Quebec, Canada) under registration number #2025-067. Participants refrained from using antibiotics, prebiotics, or probiotics for at least three months preceding the study. Participant characteristics are provided in Table S4 of the Supplementary Materials. On the day of the experiment, fresh fecal samples were collected from donors in sterile containers equipped with a GasPak™ EZ anaerobe sachet (BD Quebec, Canada) to preserve anaerobic conditions until processing.

**Figure 2:**
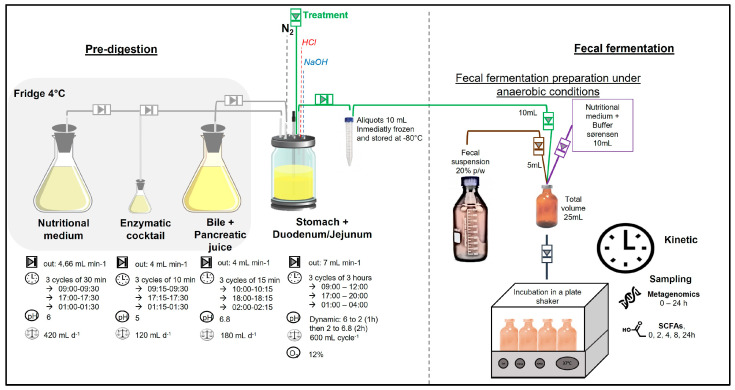
Experimental design of fecal fermentation. Acylated SCFA treatments were pre-digested in SHIME^®^, aliquoted, and stored at −80 °C. Fecal fermentation was prepared and incubated with shaking for 24 h.

The protocol was conducted in two stages:

1. Pre-digestion: To simulate gastric and intestinal phases, treatments were pre-digested in the stomach and small intestine of a simulated human intestinal model (SHIME^®^) as reported by Van de Wiele et al. [13]. Figure 2 illustrates the experimental setup, where the first vessel simulates the stomach and small intestine using a fill-and-draw method. This vessel incorporates a nutritional medium, pancreatic enzymes, and bile juice to mimic the digestive processes. Upon completion of the upper digestion phase, the vessel contents were aliquoted into 15 mL sterile tubes (10 mL) and stored at −80 °C until the fecal fermentation.
2. Fecal fermentation: A fecal suspension was prepared by mixing fresh fecal matter from each donor individually with an anaerobic phosphate buffer at a 20% (*w*/*v*) concentration, as reported by Van den Abbeele et al. [14]. In duplicate, sterile 50 mL penicillin bottles were inoculated with 5 mL of the fecal solution, 10 mL of a nutritional medium containing Sørensen buffer, and 10 mL of pre-digested aliquots. All procedures were conducted under strict anaerobic conditions. The bottles were securely sealed and maintained at a temperature of 37 ± 2 °C while shaking at 80 rpm for a duration of 24 h. This in vitro fecal batch fermentation method was standardized and validated by Lessard, et al. [15,16], to assess interindividual variability and the capacity of the GM to metabolize (poly)phenols. Time 0 samples were collected using a sterile syringe immediately after sealing the bottles. A detailed sampling schedule is provided in the Supplementary Materials.

### 2.3. Microbial Population Analysis

Samples from the batch fecal fermentation collected at 0 and 24 h were processed for DNA isolation and purification, following protocols established by Geirnaert et al. [17,18]. The DNA extracts achieved a minimum concentration of 10 ng/µL. Next-generation sequencing of 16S rRNA gene amplicon, specifically targeting the V3-V4 region (341F-805R), was conducted at the Institut de Biologie Intégrative et des Systèmes (IBIS) of Université Laval, Canada, using an Illumina MiSeq platform with Illumina V3 chemistry and a 600-cycle reagent kit (Illumina, San Diego, CA, USA) [19].

Amplicon sequence variants (ASVs) were identified using the Denoising Algorithm (DADA2) workflow, available in the dada2 R package (version 1.8) [20], which enables error modeling and correction for Illumina-sequenced amplicons (https://github.com/benjjneb/dada2 accessed on 10 October 2024). Taxonomic assignment of sequences was performed using the SILVA database [21].

### 2.4. Short-Chain Fatty Acids

Samples of 125 µL from fecal fermentation were collected according to the sampling schedule detailed in the Supplementary Materials. The fecal water was centrifuged at 14,000 rpm for 8 min at 4 °C, after which the supernatant was collected and stored at −80 °C until further processing. Before analysis, selected samples were randomized, numbered, and prepared for SCFA quantification via gas chromatography, following the method described by Roussel et al. [22].

### 2.5. Statistical Analysis

Statistical analyses and visualization of SCFAs and metagenomics data were performed using R software (version 4.3.0). Microbial community diversity during fecal fermentation was assessed via Chao and Shannon indices, with group means compared using the Kruskal–Wallis test. These calculations utilized the Vegan R package (version 2.6-4) along with the ggpubr package (version 0.6.0).

To further compare the microbiota of different donors and the effects of treatments over 24 h of fecal fermentation, distance-based redundancy analysis (db-RDA) was performed, and the significance of group separation was assessed using PERMANOVA.

Differential abundance analysis was performed using the DESeq2 package (version 1.42.0) to identify significant differences in species abundance across treatments (naringin + acetate, naringin acetate, naringin + propionate, naringin propionate, naringin, and a control). The analysis was conducted using genus-level count data and associated sample metadata. Comparisons were made between treatments at time 0 to confirm initial equivalence (no significant differences detected) and at time 24 to assess treatment effects. Sequencing depth was normalized, and dispersion was estimated using a mean-dependent fit type. Group-wise comparisons were tested using the Wald test, and resulting *p*-values were adjusted for multiple testing using the Benjamini–Hochberg procedure to control the false discovery rate (FDR). Taxa with an adjusted *p*-value < 0.05 were considered significantly differentially abundant. To improve the reliability of log2 fold change (LFC) estimates, LFC shrinkage was applied using the adaptive shrinkage estimator implemented in the ashr package [23]. Visualizations of significant results included bar plots, box plots, and volcano plots, with adjusted *p*-values highlighted according to the FDR threshold. The Wald test was used with a significant level of α < 0.05. Visualization of results included bar plots and box plots, with *p*-values calculated using the Benjamini–Hochberg post hoc test, as described by [17].

## 3. Results

### 3.1. Donors’ Colonic Microbiota Composition and Their Variability

**Figure 3:**
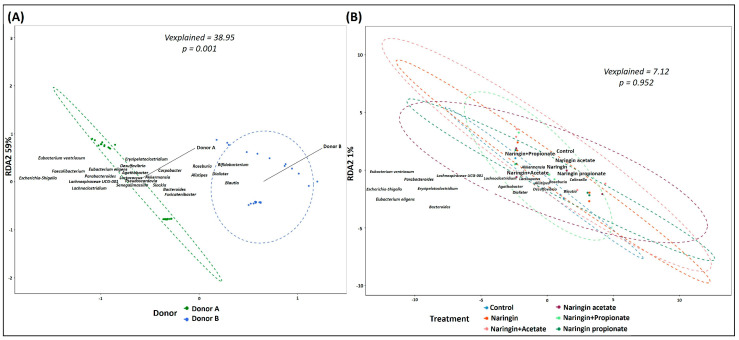
Inter-individual variability in gut microbiota composition during fecal fermentation. db-RDA of GM composition, determined by 16S rRNA gene amplicon sequencing, highlights differences between donors (inter-individual variability). RDA1 and RDA2 indicate the percentage of the total variance, whilst “Vexplained” indicates the proportion of variability and its significance, as assessed by the distance matrix PERMANOVA. Panel (**A**) illustrates individual GM profiles for both donors, while Panel (**B**) shows shifts in GM profiles over 24 h of fermentation with different treatments.

As expected, the metataxonomic analysis revealed a significant difference between the GM profiles of the two donors. Distance-based redundancy analysis (db-RDA) highlighted these distinctions, revealing that in Donor A, the most abundant genera were *Prevotella*, *Faecalibacterium*, *Blautia*, and *Lachnospiraceae*. In contrast, Donor B’s profile, in order of abundance, was characterized by *Prevotella*, *Subdoligranulum*, *Blautia*, *Faecalibacterium*, and *Bacteroides* (Figure 3A). Overall, the treatments did not affect the GM profiles, as shown in Figure 3B, where the dbRDA analysis showed no significant changes over the 24 h fermentation period for both donors (*p* > 0.05).

**Figure 4:**
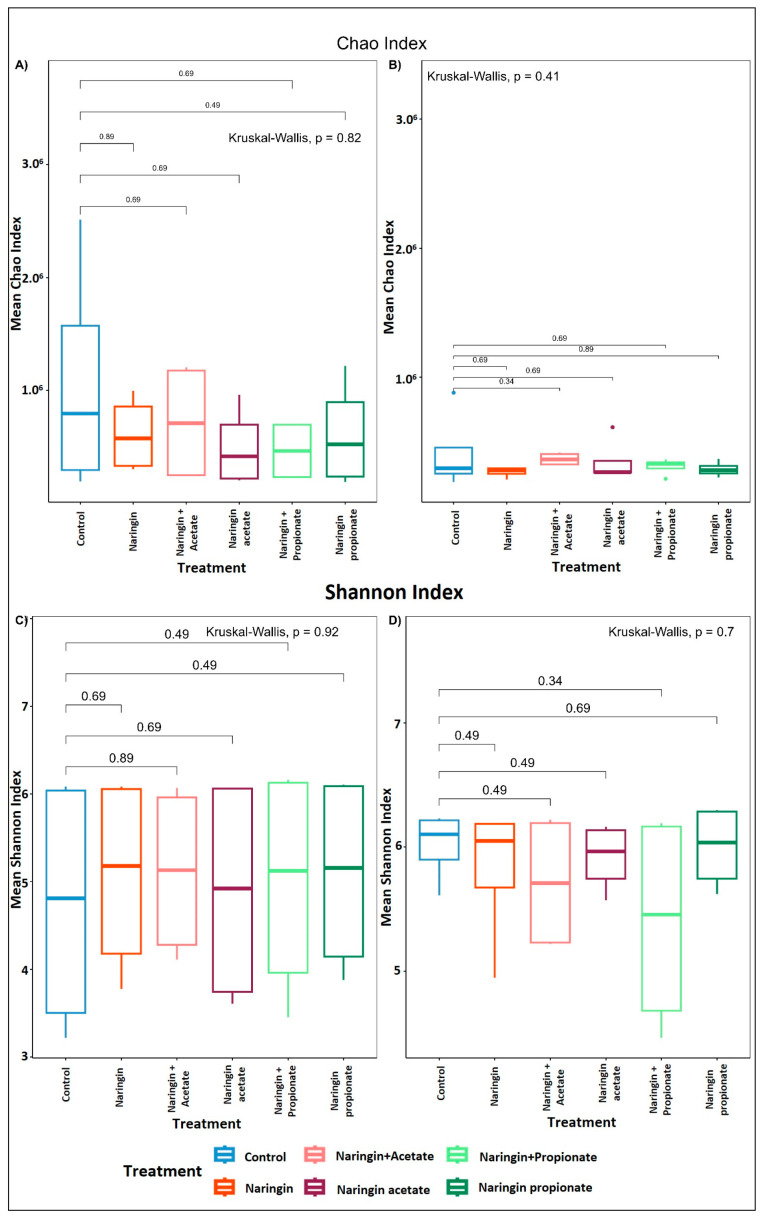
The effect of naringin treatments compared to control on the microbiota’s richness and diversity during fecal fermentation. (**A**,**B**) display richness estimated by the Chao index for donors A and B, respectively. (**C**,**D**) illustrate diversity determined by the Shannon index for donors A and B, respectively. Error bars represent SD. Significance was tested via the Kruskal–Wallis post hoc test for pairwise multiple comparisons.

The Chao and Shannon indices, which evaluate microbial diversity, richness, and species evenness (Figure 4), revealed no significant differences between treatments for any donor over the 24 h fecal fermentation period (*p* > 0.05, Kruskal–Wallis test).

**Figure 5:**
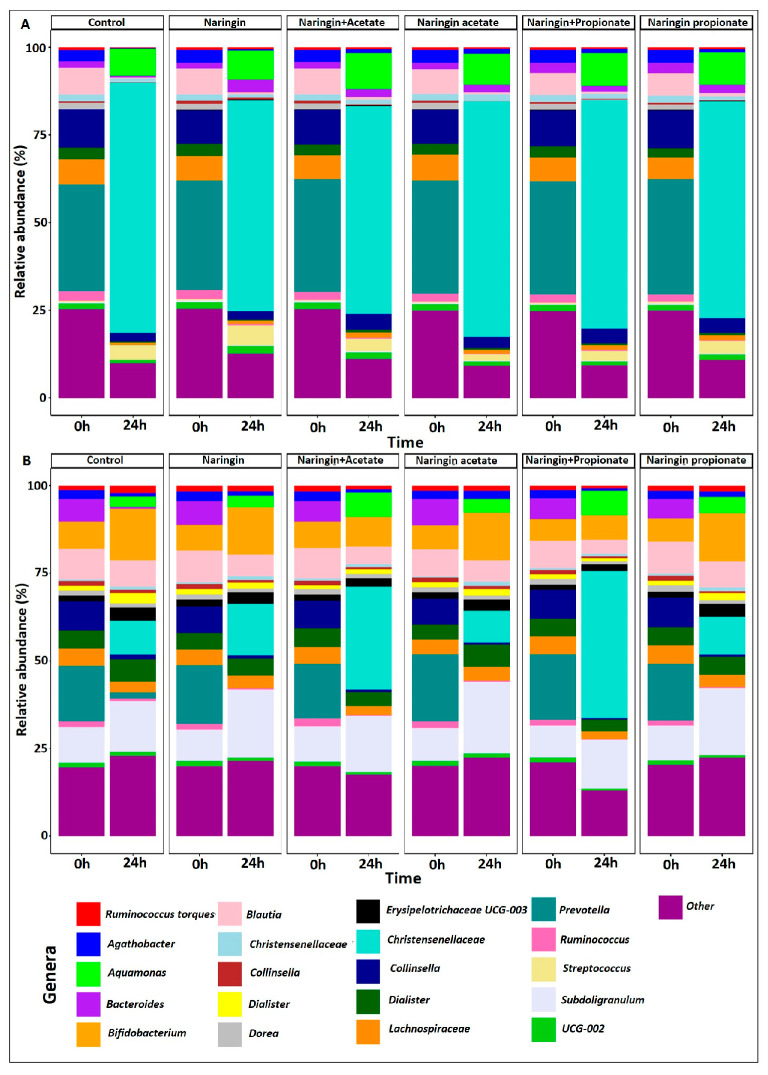
Relative abundance expressed in genera from donors’ gut microbiota during the 24 h fecal fermentation. Bar plot graphics display the relative abundance of the 20 most abundant genera in the GM of each donor at 0 and 24 h, shown separately for Donor A (**A**) and Donor B (**B**). The bar plots also illustrate the responses to various treatments (naringin acetate, naringin + acetate, naringin, naringin propionate, and naringin + propionate) compared to the control.

As illustrated in Figure 5, both donors exhibited distinct GM profiles and varying responses to the treatments. Donor A showed a dominant presence of *Escherichia*-*Shigella* after 24 h of fermentation, which remained unaffected by any treatment. In contrast, for Donor B, supplementation with naringin combined with acetate or propionate (naringin + acetate and naringin + propionate) led to more than a two-fold increase in these bacteria compared to the control (9.6%). Specifically, levels reached 29.3% and 41.9% with naringin + acetate and naringin + propionate, respectively, whereas other treatments—naringin (14.6%), naringin acetate (9.0%), and naringin propionate (10.75%)—resulted in lower levels.

In Donor A, all naringin-based treatments increased the abundance of *Bacteroides* during fermentation compared to the control, whereas this genus was nearly depleted in Donor B. Conversely, *Blautia* was better preserved during fermentation in Donor B, while it almost completely disappeared in Donor A.

Differential expression analysis revealed significant inter-donor variability in response to the various treatments. Due to the limited number of significant results, the DESeq contrasts are provided in the Supplementary Materials (Figure S1), highlighting only the significant comparisons among treatments: naringin, naringin + acetate, naringin acetate, naringin + propionate, and naringin propionate.

For Donor A, significant differences in GM composition were observed in comparisons involving naringin versus control, naringin versus naringin acetate, and naringin versus naringin + propionate. In contrast, Donor B displayed notable differences when comparing acylated and non-acylated naringin compounds, specifically between naringin acetate and naringin combined with acetate.

In Donor A, naringin supplementation resulted in only two significant shifts in bacterial abundance: *Bacteroides* increased by 2 log units (Figure S1 in Supplementary Material). When comparing naringin to naringin acetate, *Streptococcus* increased by 1.5 log units with naringin acetate, while *Agathobacter* decreased by 2 log units. Similarly, as shown in Figure S1 in Supplementary Material, the comparison between naringin and naringin + propionate revealed increases in *Collinsella*, *Bacteroides*, and *Streptococcus*, along with decreases in *Escherichia-Shigella*, *Aquamonas*, *Lachnospiraceae*, *Christensenellaceae*, *Faecalibacterium*, *Agathobacter*, and *Anaerobutyricum hallii* group. Lastly, the comparison between naringin and naringin propionate led to a 3 log unit increase in *Desulfovibrio* with naringin supplementation.

In Donor B, naringin propionate (Figure S1B) increased the abundance of *Escherichia-Shigella* and *Aquamonas*, while reducing *Bifidobacterium*, when compared to naringin + propionate treatment. Similarly, the comparison between naringin acetate and naringin + acetate (Figure S1C) revealed an increase in the *Eubacterium siraeum* group and *Escherichia-Shigella*, accompanied with a decrease in *Agathobacter* and the *Eubacterium oxidoreducens* group.

### 3.2. Increased Propionate Production with Naringin Propionate

**Figure 6:**
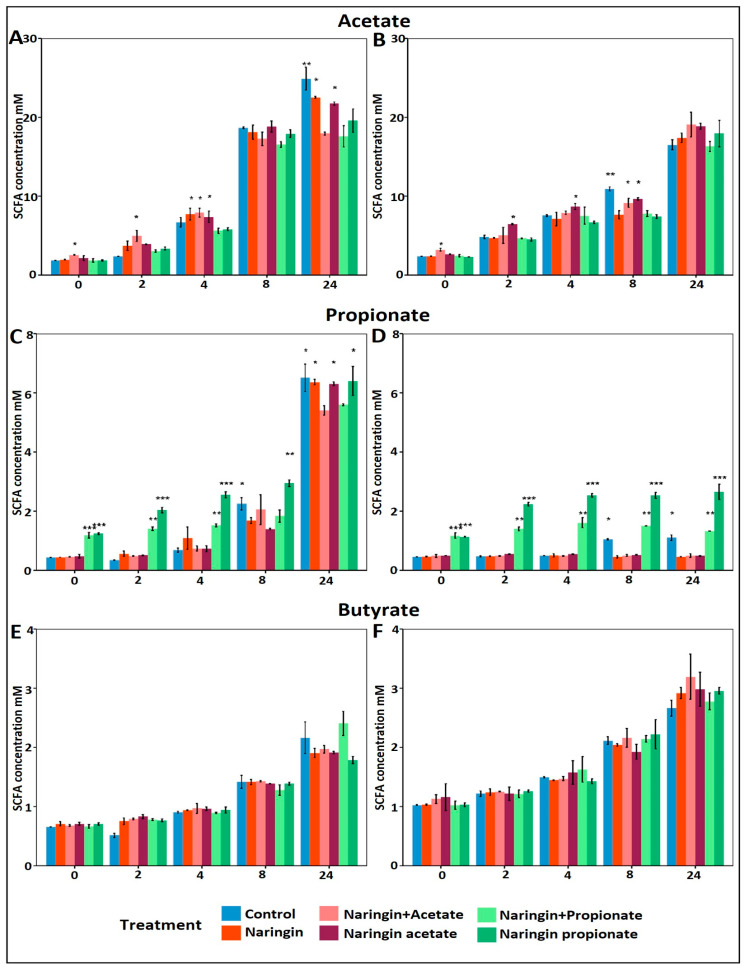
Short-chain fatty acid production during fecal fermentation. (**A**,**B**) display acetate mM concentration; (**C**,**D**) display propionate concentration; and (**E**,**F**) display butyrate mM concentration during the first 8 h of fecal fermentation by treatments. Also, (**A**,**C**,**D**) correspond to donor A, and (**B**–**D**) correspond to donor B. Significance levels are indicated as follows: * *p* = 0.05; ** *p* = 0.01; *** *p* = 0.001.

Despite the distinct microbiomes of the two donors, their baseline SCFA molar ratios were comparable: 3.6:0.8:0.5 (acetate:propionate:butyrate) for Donor A and (3.7:0.7:0.6) for Donor B. However, as illustrated in Figure 6, the dynamics of SCFA production during the first eight hours of fermentation revealed donor-specific responses to naringin-derived treatments.

Naringin and its acylated SCFA modulated microbial metabolic activity, particularly by enhancing acetate and propionate production, with limited effects on butyrate levels. Acetate production increased progressively in both donors, with Donor A (Figure 6A) exhibiting a gradual rise over 24 h across all conditions. The most substantial increases were seen within the first four hours in treatments containing supplemental acetate (naringin acetate and naringin + acetate). At time 0, the naringin acetate already induced a 0.26 mM increase over the control, while the naringin + acetate yielded a larger 0.75 mM rise over to the control.

Propionate concentrations followed a similar trend. Treatments enriched with propionate (naringin propionate and naringin + propionate) significantly elevated propionate levels in both donors (Figure 6C,D). The strongest and most sustained stimulation occurred in the naringin propionate treatment group (*p* < 0.0001), starting at hour 2 and persisting throughout the fermentation. Furthermore, both donors exhibited a slight plateau in propionate levels up to 8 h; however, only donor A showed a highly significant increase in propionate towards the end of fermentation across all experimental conditions. Notably, naringin propionate led to significantly higher levels of propionate compared to an equimolar amount of naringin + propionate in both donors. At time 0, the naringin propionate group already displayed a 0.74 mM increase over the control, while the naringin + propionate group showed a comparable increase of 0.75 mM.

By contrast, butyrate production remained largely unaffected by all treatments in both donors (Figure 6E,F). However, butyrate levels gradually increased over the fermentation period, indicating a time-dependent effect rather than a response specific to the treatment or donor.

### 3.3. Distinct Microbial Shifts Induced by Naringin Treatments Highlight Limited but Specific Changes in GM Composition

**Figure 7:**
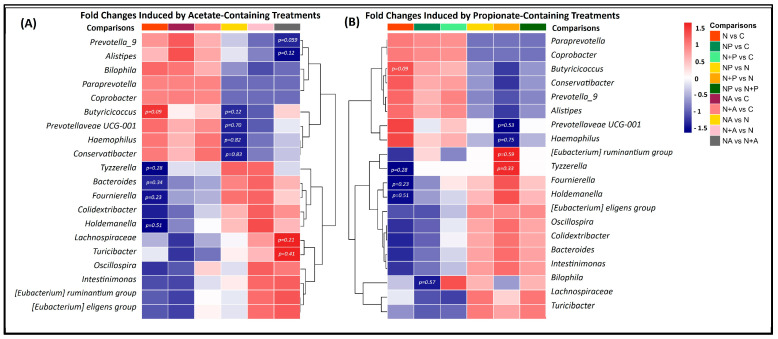
Heat map displaying DESeq-based comparisons of GM composition in response to naringin treatments, highlighting tendencies in differential abundance across taxa. Panel (**A**) shows the changes induced by acetate-containing treatments versus controls, while Panel (**B**) shows the changes induced by propionate-containing treatments versus controls. Treatment comparisons are labeled as follows: N vs. C = naringin versus control, NP vs. C = naringin propionate versus control, N + P vs. C = naringin + propionate versus control, NP vs. N = naringin propionate versus naringin, N + P vs. N = naringin + propionate versus naringin, NP vs. N + P = naringin propionate versus naringin + propionate, NA vs. C = naringin acetate versus control, N + A vs. C = naringin + acetate versus control, NA vs. N = naringin acetate versus naringin, N + A vs. N = naringin + acetate versus naringin, NA vs. N + A = naringin acetate versus naringin + acetate.

Figure 7 summarizes the top 20 trending genera identified through DESeq analysis across all treatment conditions, relative to naringin acetate (Figure 7A) and naringin propionate (Figure 7B), in both donors, complementing the results presented in the previous section. These genera were selected for their consistent presence across treatments, despite *p*-values exceeding 0.05, as their log2 fold changes ranged between −1.5 and 1.5.

Naringin-based treatments (naringin, naringin acetate, and naringin + acetate) were associated with an increased abundance of genera such as *Prevotella 9*, *Alistipes*, *Bilophila*, *Coprobacter*, *Butyricicoccus*, *Prevotellaceae UCG-001*, *Hemophilus*, and *Conservatibacter*. In parallel, these treatments were linked to a reduction in several taxa, including *Tyzerella*, *Bacteroides*, *Fournierella*, *Colidextribacter*, *Holdemanella*, *Lachnospiraceae*, *Turicibacter*, *Oscillospira*, *Intestinimonas*, *Eubacterium ruminatium*, and *Eubacterium eligens*, compared to the untreated control.

When comparing naringin acetate and naringin + acetate to naringin alone, there was an increased relative abundance of *Tyzzerella*, *Bacteroides*, *Fournierella*, *Colidextribacter*, and *Holdemanella*, while *Butyricicoccus*, *Prevotellaceae UCG-001*, *Hemophilus*, and *Conservatibacter* were reduced. Interestingly, comparisons between naringin and acetate treatments showed that naringin acetate strongly stimulates *Lachnospiraceae* and *Turicibacter*, and reduces *Prevotella* and *Alistipes*, compared to naringin + acetate.

As shown in Figure 7B, treatments combining naringin and propionate (naringin propionate and naringin + propionate) led to an increase in *Fournierella*, *Holdemanella*, *Eubacterium eligens*, *Oscillospira*, *Colidextribacter*, *Bacteroides*, *Intestinimonas*, *Lachnospiraceae*, and *Turicibacter*, and a decrease in *Paraprevotella*, *Coprobacter*, *Butyricicoccus*, *Conservatibacter*, *Prevotella 9*, *Alistipes*, and *Haemophilus*, compared to naringin alone. Finally, the comparison between the acylated compound (naringin propionate) and naringin + propionate revealed an increase in *Fournierella*, *Holdemanella*, *Eubacterium eligens*, *Oscillospira*, *Colidextribacter*, *Bacteroides*, *Intestinimonas*, *Bilophila*, *Lachnospiraceae*, and *Turicibacter* and a decrease in *Paraprevotella*, *Coprobacter*, *Butyricicoccus*, *Conservatibacter*, *Prevotella 9*, *Alistipes*, and *Haemophilus.*

Overall, all naringin treatments, including the “parent” compound (naringin alone), co-supplemented forms (naringin + acetate and naringin + propionate), and the acylated compounds (naringin propionate and naringin acetate), were associated with a general enrichment of *Prevotella 9*, *Alistipes*, *Coprobacter*, *Butyricicoccus*, *Prevotellaceae UCG-001*, *Hemophilus*, and *Conservatibacter*, alongside a reduction in *Fournierella*, *Colidextribacter*, *Holdemanella*, *Lachnospiraceae*, *Turicibacter*, *Oscillospira*, *Intestinimonas*, *Eubacterium ruminatium*, and *Eubacterium eligens.* Donor-specific comparisons are available in the Supplementary Materials (Figures S2 and S3).

## 4. Discussion

Recent findings indicate that SCFAs are essential for immune regulation and gut integrity. Propionate and butyrate, in particular, play key roles in reducing inflammation, supporting immune function, and maintaining mucosal homeostasis, yet their synthesis is compromised by low dietary fiber intake, antibiotic treatments, and dysbiosis [5,7]. Enhancing SCFA levels in the intestine is therefore a promising strategy for preserving mucosal homeostasis and gut health, especially in conditions where fiber deficiency disrupts microbial fermentation and SCFA synthesis [5,7].

Various approaches have been proposed to enhance the SCFA levels in the gut, including increased dietary fiber and prebiotic intake, probiotic supplementation, exogenous SCFA administration, and the development of controlled-release SCFA formulations. Dietary fiber and prebiotics stimulate microbial fermentation, leading to the production of SCFAs as key metabolic byproducts [24,25]. According to the International Scientific Association for Probiotics and Prebiotics, recent evidence also supports the ability of probiotics to enhance SCFA production [26], particularly next-generation probiotics selected for their capacity to boost specific SCFAs. For example, *Faecalibacterium prausnitzii* and *Clostridium butyricum* are closely associated with butyrate production and confer additional benefits such as anti-inflammatory and antidiabetic effects [27]. However, exogenous SCFA oral supplementation is limited by the rapid absorption of the SCFAs in the upper gastrointestinal tract via passive diffusion, thereby reducing their delivery to the colon [28,29]. To address this limitation, several strategies have been developed to enable targeted delivery and controlled release of SCFAs directly in the colon. These include the use of digestion-resistant carriers, SCFA-acylated molecules [11,12,30], and encapsulated SCFAs [31]. Among these, digestion-resistant carriers containing SCFAs or SCFA-acylated molecules offer multiple benefits, serving as both a direct SCFA source and a potential prebiotic, depending on the carrier molecule used.

In this context, our research group has developed innovative SCFA-acylated naringin derivatives from citrus co-products, designed to enhance the targeted delivery of SCFAs within the gastrointestinal tract [12]. These derivatives consist of propionate or acetate conjugates acylated to the glycosylated moiety of naringin (Figure 1). This study evaluated the potential of naringin propionate to function as an exogenous source of propionate and assessed whether naringin acetate could promote butyrate production through cross-feeding mechanisms. Additionally, we investigated the capacity of these molecules to modulate GM composition and promote naringin bioavailability through microbial deglycosylation. To address these questions, we conducted a two-stage, 24 h in vitro batch fecal fermentation using fecal inoculum from two donors.

Acylated naringin derivatives promoted a sustained release of SCFAs during fermentation. Our results showed that both donors exhibited similar trends in SCFA production and release, with a slight increase in acetate and strong significant increases in propionate following treatments. Acetate increased up to 4 h of fermentation for exogenous sources of acetate, while propionate increased up to 8 h, especially with the acylated form (naringin propionate) compared to the combination of naringin and free propionate. These findings suggest that microbial hydrolysis of the acyl group augmented SCFA production beyond baseline levels generated through normal fermentation processes. Previous studies have reported that esterified forms of SCFAs can enhance delivery and release in the colon. For instance, Chambers et al. demonstrated that inulin esterified with propionate improved colonic SCFA delivery and glucose homeostasis [11]. Likewise, Wang et al. and Tain et al. reported that tyrosol and resveratrol-esterified SCFAs significantly increased SCFA concentrations in target tissues, enhancing physiological outcomes [30,32]. These findings support the use of naringin-acylated derivatives as promising tools for delivering SCFAs to the colon in a sustained and controlled manner.

Initial differences in acetate levels reflect the release dynamics of acylated treatments. At the onset of fermentation, acetate concentrations were comparable across all experimental conditions, except in the naringin + acetate group—an anticipated result, which showed a significant increase of 0.75 mM compared to the control (*p* < 0.05). In contrast, the naringin acetate group exhibited only a small, non-significant increase of 0.26 mM. Over the first four hours, acetate levels in the naringin acetate group gradually increased, likely reflecting the time required for GM to initiate hydrolysis of the acylated compound. Studies on hydroxytyrosol- and tyrosol-SCFA esters have shown that GM mediates their hydrolysis, leading to a structure-dependent, time-controlled release of SCFAs [32]. These findings support the notion that acylated derivatives like naringin acetate can provide a delayed release of SCFAs, contributing progressively to the SCFA pool during fermentation.

Propionate release was enhanced and prolonged following naringin propionate treatment. At time zero, both naringin propionate and naringin + propionate treatments showed significantly higher propionate levels compared to the control, with differences of 0.74 mM and 0.75 mM, respectively. While a higher concentration was initially expected only in the naringin + propionate treatment, the early release in the naringin propionate group may be due to possible spontaneous hydrolysis of the acyl bond. However, from hour 2 onward, propionate concentrations were consistently higher with the acylated derivative, indicating sustained release. This behavior has been demonstrated in other esterified systems. For example, the use of esterified SCFA with dietary compounds has shown promise in achieving targeted delivery, as reported by Chambers et al., Wang et al., and Tain et al. [11,30,32]. These studies emphasize the value of acylated derivatives in enhancing SCFA bioavailability in the colon. Accordingly, our findings support the concept that acylation of naringin is an effective strategy to improve propionate delivery and maintain elevated levels over time during colonic fermentation.

Interestingly, butyrate levels remained unchanged despite increased acetate availability from naringin acetate or from the addition of acetate. Although treatments included naringin acetate and added acetate, butyrate concentrations remained unchanged through the fermentation process. This outcome contradicts our initial hypothesis that increased acetate would stimulate butyrate production through the Butyryl-CoA: acetate CoA-transferase pathway. Previous studies have described this metabolic pathway as a key mechanism by which certain gut bacteria convert acetate into butyrate [25,26]. Our findings suggest that, under the present experimental conditions, this cross-feeding interaction did not take place.

Acetate produced in fecal fermentation may have been utilized for alternative metabolic pathways. Instead of being converted into butyrate, acetate released from acylated naringin may have been taken up by bacterial cells and rerouted to support other essential biosynthetic functions. These could include amino acid synthesis or intermediary metabolism via the TCA cycle. Wolfe (2005) described how bacterial cells often prioritize acetate for vital cellular processes, depending on their metabolic demands and environmental conditions [33]. Therefore, it is plausible that acetate availability alone was insufficient to initiate butyrate synthesis due to competing cellular metabolic priorities.

The limited duration of fermentation may have constrained cross-feeding and butyrate production. A 24 h fermentation period may not have allowed sufficient time for the microbial community to adapt and establish syntrophy relationships essential for effective cross-feeding, particularly the conversion of acetate to butyrate. Teichmann and Cockburn (2021) highlighted that short-term batch fermentations often impede microbial cooperation and nutrient sharing, with an inadequate accumulation of cofactors like vitamins to support butyrate-producing species such as *Rumminococcus bromii* [34]. Nevertheless, a 24 h incubation was selected to simulate the physiological conditions of the proximal colon, which typically has a retention time of 30–40 h in healthy individuals according to Arhan et al. and Tomita et al. [35,36]. This duration aimed to balance ecological relevance with the experimental control while avoiding the accumulation of toxic by-products that could emerge during extended fermentation.

Naringin’s antibacterial and iron-chelating properties may have negatively impacted butyrate production by altering microbial community structure. In our study, key butyrate-producing bacteria taxa—*Faecalibacterium*, *Lachnospiraceae*, and *Ruminococcus*—were significantly reduced in all naringin-treated groups compared to the control. This effect may result from naringin’s dual mechanism: disrupting bacterial populations and limiting iron availability in the gut, an essential factor for the growth of many butyrate producers. Given that these taxa are particularly sensitive to iron concentrations, their diminished abundance could explain the lower butyrate levels observed. Previous studies have documented naringin’s antimicrobial activity [37] and its ability to chelate iron [38], both of which are especially relevant to iron-sensitive butyrate-producing bacteria [39,40]. However, other contributing factors should also be considered to explain this response. For instance, insufficient enzymatic activity, low functional redundancy among butyrogenic taxa, or an initial underrepresentation of cross-feeding species capable of converting acetate to butyrate may have limited butyrate formation. Additionally, the static nature of the batch fermentation and the short 24 h incubation may not allow sufficient time for the establishment of cross-feeding interactions or the full expression of metabolic pathways, such as the acetate-to-butyrate conversion, which typically require longer adaptation periods. On the other hand, in the case of naringin propionate, although chelation could still occur, the hydrolysis of the acylated compound in the colon allowed effective propionate release. This result aligns with findings from Wang et al. and Tain et al., who demonstrated the efficient delivery of propionate to the colon through esterified compounds [30,32]. Collectively, these findings support the idea that naringin propionate can enhance colonic propionate concentrations despite microbial shifts, while the iron-chelating properties of naringin may limit the metabolic niche of butyrate producers and restrict acetate-to-butyrate conversion.

Contrary to expectations, the combination of naringin and SCFAs induced significant changes in GM composition. Despite the anticipated benefits, such as enhanced proliferation of probiotic bacteria and reduced abundance of potentially pathogenic taxa, our results showed limited significant shifts in the GM community following these treatments. This outcome may be influenced by several factors known to affect diversity responses in prebiotic studies, including the experimental setup, the number of gut microbes capable of utilizing the compound, and their initial relative abundances, as highlighted by Cantu-Jungles and Hamaker [41]. When a bioactive compound is metabolized (e.g., fiber) by multiple taxa, the energy derived from it is distributed among many bacteria, potentially diluting its impact on the growth of individual species and, consequently, on alpha diversity metrics such as evenness. Previous studies have demonstrated that exogenous SCFA supplementation can significantly modulate GM and support immune function. For instance, Zajac et al. demonstrated that a five-month SCFA treatment increased alpha diversity and promoted the growth of SCFA-producing bacteria in mice [42]. Similarly, a two-week supplementation with an SCFA mixture enhanced the abundance of beneficial taxa such as *Akkermansia muciniphila* and *Escherichia fergusonii* [43]. Bell et al. found that a six-week supplementation with high-amylose maize-resistant starch modified with acetate and butyrate elevated SCFA levels in stool and plasma and altered GM composition in adults with type 1 diabetes, potentially improving immune tolerance and glycemic control [44]. However, the absence of significant changes in alpha diversity in our study does not necessarily imply a lack of ecological impact. As highlighted by Dong et al., phenolic compounds such as those in oats can exert meaningful functional and compositional effects on the microbiota without significantly altering overall diversity indices [45]. In our case, although Chao and Shannon indices remained stable, the naringin SCFA treatments led to increase propionate levels and modulate specific taxa associated with flavonoid metabolism, suggesting a more targeted form of microbial modulation.

Although we anticipated a more pronounced modulation of GM by naringin and its derivatives, the observed changes were modest after 24 h of fermentation. In contrast to previous findings by Cao et al. and Van Rymenant et al. [46,47], our results showed only moderate shifts in GM composition following naringin treatments. As shown by DESeq’s significant changes (Figure S1), Donor A exhibited an increase in *Bacteroides*, *Streptococcus*, and *Oscillospiraceae* UCG-002, while Donor B showed a selective enrichment in *Eubacterium siraeum*. Notably, non-acylated naringin significantly increased the relative abundance of *Bacteroides.* This shift is relevant since *Bacteroides* is considered a keystone taxon involved in maintaining microbial community structure, modulating SCFAs production [48,49,50], and producing antimicrobial compounds that inhibit competing bacteria [51]. Thus, even modest changes, including the increase in *Bacteroides*, may have favorable implications for gut health.

In line with these findings, a DESeq analysis revealed trends across all naringin treatments, particularly promoting genera linked to gut health and key metabolic functions. An increase in *Prevotella 9* and *Prevotellaceae UCG-001*—genera commonly associated with high-fiber diets, efficient carbohydrate fermentation, and the production of SCFAs such as acetate and succinate [52,53], which can impact host energy metabolism and gut barrier function—was observed. Previous studies reported similar effects, with *Prevotellaceae* abundance rising in response to flavonoid treatments such as kaempferol [54], puerarin [55], hesperetin-7-O-glucoside, rutin, and isoquercitrin [56]. However, while *Prevotella* abundance has been associated with improved glucose metabolism in some dietary contexts, excessive succinate accumulation may also have pro-inflammatory consequences [57]. Additionally, *Prevotellaceae* may exert a protective effect neutralizing reactive oxygen species and alleviating intestinal oxidative stress, as reported by Chen et al. in the context of inflammatory bowel disease (IBD) [58]. Changes in *Bacteroides* were also observed and are functionally relevant. These species are known for their versatility in degrading complex polysaccharides and for their production of propionate and acetate, which have been associated with the modulation of immune function and the maintenance of gut barrier integrity [59,60]. The balance between *Bacteroides* and *Prevotella* may reflect broader enterotype dynamics, influencing SCFA profiles and overall host physiology. By modulating the relative abundance of these key taxa, SCFA-acylated naringin derivatives may affect not only microbial composition but also functional outputs with potential implications for host metabolic health. Notably, *Alistipes*, another genus linked to weight regulation and metabolic health [61,62], was depleted across treatments, in line with its reported association with inflammation. Moreover, naringin treatments appeared to negatively impact certain beneficial butyrate-producing bacteria, such as *Intestinimonas* [63] and *Eubacterium ruminantium* [64], suggesting a potential trade-off in microbial functional composition. Future studies using metagenomic or metabolomic approaches will be essential to clarify the consequences of these microbial shifts on functional outcomes.

Overall, these microbial shifts are consistent with previously reported mechanisms of action for flavanols like naringin. For instance, Van Rymenant et al. showed that a three-week treatment with 500 mg of citrus extract rich in hesperidin and naringin altered SCFA production and reduced the relative abundance of *Clostridium coccoides*/*Eubacterium rectale* [47]. Similarly, Pakar et al. reported that naringin promoted *Lactobacillus rhamnosus* while inhibiting *Escherichia coli* and *Salmonella typhimurium*, with *Staphylococcus aureus* being more sensitive at a minimal concentration of 62.5 µg/mL [65]. Celiz et al. further demonstrated the strong antibacterial activity of naringin metabolites against Gram-positive strains such as *L. monocytogenes* and *S. aureus* [37]. In our study, naringin also exerted antimicrobial effects on *Agathobacter* (Firmicutes), as well as *Aquamonas* (Proteobacteria), although no inhibitory activity was noted against *Escherichia-Shigella*. We speculate that *Escherichia-Shigella*, being saccharolytic, may have thrived in the carbohydrate-rich culture medium, an effect that is addressed below.

Acetate-enriched naringin treatments modulated GM by promoting genera associated with metabolic function and immune regulation. Compared to naringin alone, both the naringin acetate and naringin + acetate treatments showed a trend toward slightly increased abundances of *Bacteroides*, *Fournierella*, and *Holdemanella*—genera implicated in short-chain fatty acid production and metabolic activity. However, these differences did not reach statistical significance. *Bacteroides* contributes to acetate and propionate synthesis through the fermentation of complex carbohydrates [63]; *Fournierella* is an established acetate producer [66]; and *Holdemanella* has been linked to metabolic regulation [67]. Additionally, *Colidextribacter* and *Holdemanella*, both associated with tumor-suppressive effects in murine and human studies [68,69], were more abundant in the acetate-supplemented groups. When comparing naringin acetate to naringin + acetate, a marked increase in *Lachnospiraceae* and *Turicibacter* was observed: the latter is known for modulating host bile acid profiles and lipid metabolism [70]. However, these treatments also favored the proliferation of *Tyzzerella*, a genus linked to pro-inflammatory responses and obesity [71]. At the same time, there was a reduction in the relative abundance of beneficial genera such as *Butyricicoccus* (a butyrate producer [72]), *Prevotellaceae UCG-001* (a propionate producer [73]), and *Haemophilus*, a commensal bacterium associated with mucosal health but also IBD [74]. These observations suggest that acetate co-supplementation with naringin can enhance certain health-promoting microbial taxa while also inducing shifts that may have pro-inflammatory or pathogenic potential, warranting further investigation.

Propionate-enriched naringin treatments modulated the GM by enhancing the abundance of genera in carbohydrate fermentation and (poly)phenol metabolism. Compared to naringin alone, fermentation with naringin propionate and naringin + propionate led to increased levels of *Eubacterium eligens*, *Bacteroides*, and *Lachnospiraceae FD2005*-genera known for their capacity to metabolize polysaccharides [75,76,77] and (poly)phenols [78]. *Oscillospira*, a genus shown to increase in response to dietary carrot (poly)phenols [79], was also enriched. Interestingly, a trend toward increased abundance of several beneficial genera was observed with naringin propionate treatment, relative to naringin + propionate. Naringin propionate induced an increase in several beneficial genera, including *Fournierella*, *Holdemanella*, *Eubacterium eligens*, *Oscillospira*, *Colidextribacter*, *Bacteroides*, *Intestinimonas*, *Bilophila*, *Lachnospiraceae*, and *Turicibacter*, compared to naringin + propionate.

The unexpected expansion of *Escherichia-Shigella* across all groups highlights the influence of external factors such as medium composition on GM responses. In our study, *Escherichia-Shigella* increased in abundance in both treatment and control groups, with a more pronounced expansion in donor A. Donor B, however, showed a stronger response specifically to the non-acylated naringin treatments (naringin + acetate and naringin + propionate). This trend appears to be driven primarily by the composition of the SHIME^®^ nutritional medium, which is rich in mono- and polysaccharides and other nutrients that support *Escherichia-Shigella* growth. Yousi et al. showed that this genus thrives in environments with similar nutrient profiles, including pectin, xylan, arabinogalactan, yeast extract, bile salts, and mucin [80]. SCFAs are known to modulate GM dynamics and host–pathogen interactions by modulating intestinal pH, GM composition, and intestinal barrier integrity. However, direct evidence linking exogenous SCFA supplementation to *Escherichia-Shigella* remains unexplored [81]. Furthermore, the overrepresentation of *Escherichia-Shigella* in our system underscores a broader limitation of in vitro models, such as static batch fermentation or SHIME^®^, which lack immune responses, mucosal barriers, and host-derived signals that normally regulate microbial populations in vivo. As noted by Venema and van den Abbeele [82] and Li et al. [83], such discrepancies may lead to an ecological disconnect between in vitro conditions and physiological environment, complicating the interpretation of treatment-specific effects. Taken together, these findings suggest that the observed proliferation of *Escherichia-Shigella* is likely attributed more to the growth-supportive properties of the medium than to the specific effects of SCFAs or naringin supplementation.

Naringin supplementation promotes the growth of bacteria capable of flavonoid deglycosylation, irrespective of SCFA acylation. All treatments containing naringin led to an increase in bacteria known to express deglycosylation enzymes, regardless of the presence or absence of the SCFA acyl groups. *Agathobacter*, which was enriched across treatments, has been reported to produce flavonoid deglycosylation enzymes [84]. Likewise, several species within *Bacteroides* (e.g., *B. uniformis* and *B. distasonis*) and *Streptococcus* (*S. faecalis* and *S. lactis*) exhibit O-deglycosylation activity [85,86]. *Eubacterium* species also showed notable increases, including *Eubacterium siraeum*, which carries a flavonoid degradation pathway according to the KEGG database [87], and *E. ramulus*, previously identified as having O-deglycosylation capability [88]. Although *Oscillospiraceae* has not been directly linked to flavonoid degradation enzymes, multiple studies reviewed by Yang et al. support a positive association between (poly)phenol intake and this genus, which is also proposed as a next-generation probiotic [89]. Additionally, *Eubacterium oxidoreducens*, observed in higher abundance, is known to degrade flavonoids such as gallate, pyrogallol, phloroglucinol, and quercetin [90]. *Bifidobacterium*, *Streptococcus*, and *Bacteroides* were also shown to metabolize flavonoids such as eriocitrin in vitro [86], with various strains of *Bifidobacterium* expressing glycosylases and other enzymes involved in the breakdown of oligosaccharides and glycol-conjugated molecules [91,92,93,94,95]. Together, these results suggest that naringin favors the proliferation of flavonoid-degrading bacteria, potentially enabling them to access glycosyl groups as an energy source—a hypothesis that warrants further investigation.

The deacylation of SCFA-acylated naringin by the GM may occur independently of deglycosylation, facilitating efficient SCFA release. In SCFA-acylated naringin derivatives, SCFAs are bound to the glucosyl group, suggesting that the GM may have deacylated the molecule to access glucose, thereby releasing SCFAs in the process. Supporting this mechanism, microbial esterases—often expressed at the bacterial cell surface—are known to catalyze deacylation efficiently, without requiring cofactors for activity [96,97,98,99]. In contrast, deglycosydases are primarily secreted into the extracellular medium or anchored to the cell membrane, where they target complex carbohydrates and flavonoids [100]. This functional distinction suggests that SCFA naringin could have been deacylated without simultaneous cleavage of the glucosyl moiety. Although a notable increase in acetate and propionate concentrations was observed in the fermentation medium, the relative abundance of deglycosylating bacteria did not show a comparable increase. These observations indicate that GM possesses the enzymatic capability to deacylate SCFA-acylated naringin independently of deglycosylation, potentially enhancing SCFA bioavailability in the colon.

While our in vitro fecal fermentation model provides some insights into the modulatory effects of SCFA-acylated naringin derivatives on GM composition and SCFA levels, translating these findings into in vivo contexts requires careful consideration. Factors such as compound stability during gastrointestinal transit, absorption, metabolism, and effective delivery to the colon must be addressed. To date, only oxidative stability has been evaluated [12], but these compounds may undergo enzymatic degradation in the upper gastrointestinal tract, potentially affecting their activity [101,102]. Given the generally low bioavailability of flavonoids and the key role of microbial metabolism in their effects [103], the effectiveness in vivo dose may differ from in vitro concentrations, as William and Clifford reported [104]. Atta et al. highlighted microencapsulation as a strategy to enhance targeted treatments [105]. Similarly, formulation strategies may be needed to ensure the stability and colon-specific release of SCFA-acylated naringin. In vivo studies, including pharmacokinetic profiling and functional assessments, will be essential to validate these findings and confirm the prebiotic potential of these compounds.

This study presents several methodological limitations that should be considered when interpreting the findings. Key limitations include the small number of GM donors, the relatively short fermentation duration, and the use of static batch fermentation. In vitro models, valuable for controlled mechanistic investigations, cannot fully replicate the complexity of host–microbiota interactions, observed in in vivo studies, such as immune responses, mucosal adhesion, and longitudinal community dynamics, as highlighted by Li et al. [83]. These factors limit the ability to capture bidirectional host–microbiota communication, colonization patterns, and time-dependent microbial shifts influenced by host metabolism and immune modulation. Yet, they are acceptable when the objective is to assess the dynamic of SCFA release from SCFA-acylated as in our case. Previous studies have emphasized the importance of including a larger number of donors to better capture interindividual variability and reflect the diversity of human GM [16]. Nonetheless, despite using only two donors with contrasting microbial profiles, our study observed directional changes in key functional metrics, including SCFA production and metagenomic responses, supporting the reproducibility of the observed effects. For instance, both donors exhibited similar trends in SCFA levels throughout the fermentation period. Furthermore, this study was designed as a proof-of-concept confirming the concept that SCFA-acylated naringin derivatives can enhance SCFA concentrations in the colon. The fermentation duration also plays a critical role in shaping microbial activity and metabolite output. However, extending fermentation beyond 24 h can lead to the accumulation of toxic secondary metabolites, which may inhibit microbial growth and distort metabolic responses (including cross-feeding). For instance, Osborne et al. showed that prolonged batch fermentation of *Escherichia coli* resulted in the buildup of volatile toxic compounds, adversely affecting cell viability and metabolic activity [106]. On the other hand, Pirkola et al. observed only modest treatment-related differences over a 24 h fermentation [107]. To improve future study design, the use of semi-dynamic fermentation or chemostat fermentation systems could help minimize metabolite accumulation and more accurately reflect the dynamic interactions between GM and dietary compounds over time. Despite the noted limitations, our study provides a valuable preliminary framework that can inform future research and guide methodological refinements in the exploitation of microbiota–metabolite interactions.

## 5. Conclusions

The present study highlights the potential of acylated naringin compounds to enhance the release of SCFAs, particularly propionate and acetate, during in vitro fecal fermentation. Naringin propionate emerged as a promising vehicle for colonic propionate delivery. However, the lack of impact on butyrate suggests limited cross-feeding activity. Although microbial diversity changes were minimal, certain treatments showed tendencies to influence health-associated microbial populations, fermentation-related taxa, and bacteria with deglycosylation capabilities with donor-specific variations. Overall, these findings underscore the potential of acylated naringin as a functional dietary supplement for modulating GM and enhancing SCFA release. Despite the methodological limitations of this study, the results provide valuable insights into the effects of naringin and its derivatives on GM, offering a framework for future research. Additionally, the potential of leveraging a technological process that utilizes citrus co-products to modulate the gut microbiota and potentially mitigate metabolic diseases is highlighted. Further research is necessary to confirm these observations in an in vivo preclinical or clinical setting.

## Figures and Tables

**Table 1:** Chemical composition of naringin acyls compounds.

Compound	Molecular Weight	%SCFAs	%Naringin	Naringin (g)	SCFAs (g/Mole)
Naringin acetate	621.0	9.1	90.9	564	56.51
Naringin propionate	635	11.5	88.5	562	73.03
Naringin	580.5	-	100.0	-	-

For the naringin acetate and naringin propionate controls, the concentrations of naringin and acetate or propionate (2.6 mM) added were equivalent to those in their respective individual treatments.

## Data Availability

Supporting information can be downloaded at https://www.ncbi.nlm.nih.gov/bioproject/PRJNA1267726 (accessed on 25 May 2025).

## Supplementary Materials

The following supporting information can be downloaded at: https://www.mdpi.com/article/10.3390/life15060967/s1, Figure S1: DESeq Analysis of GM: Treatment comparisons; Figure S2: Heat map displaying DESeq-based comparisons of GM composition in response to Naringin treatments, highlighting tendencies in differential abundance across taxa in Donor A; Figure S3: Heat map displaying DESeq-based comparisons of GM composition in response to Naringin treatments, highlighting tendencies in differential abundance across taxa in Donor B; Table S1: Anaerobic buffer composition; Table S2: Nutritional SHIME^®^ medium composition modified; Table S3: Pancreatic juice composition; Sampling schedule; Table S4: Characterization of the donors.

## Abbreviations

The following abbreviations are used in this manuscript:

NCDs	Non-communicable diseases
GM	Gut microbiota
SCFAs	Short-chain fatty acids
IBD	Inflammatory bowel disease
SHIME	Simulator of the Human Intestinal Microbial Ecosystem
g	Gram
M	Molar
mL	Milliliter
w/v	Weight per volume
°C	Degrees Celsius
rpm	Revolutions per minute
DNA	Deoxyribonucleic acid
ng	Nanogram
µL	Microliter
rRNA	Ribosomal ribonucleic acid
ASVs	Amplicon sequence variants
db-RDA	Distance-based redundancy analysis
DESeq	Differential expression analysis based on Negative Binomial distribution
Log	Logarithm
TCA	Tricarboxylic acid
RDA	ReDundandy Analysis
SD	Standard deviation
