# Supplementary material for: Novel Acylated Naringin Enhances Propionate Release and Stimulates the Growth of Flavanone-Metabolizing Bacteria in an In Vitro Batch Fermentation Model
Source: Life (Basel). 2025 Jun 17;15(6):967. doi: 10.3390/life15060967 (PMC12193867; doi:10.3390/life15060967)
Supplement: Supplementary file 1 [file life-15-00967-s001.zip › life-3657123-supplementary.pdf]

SUPPLEMENTARY MATERIAL

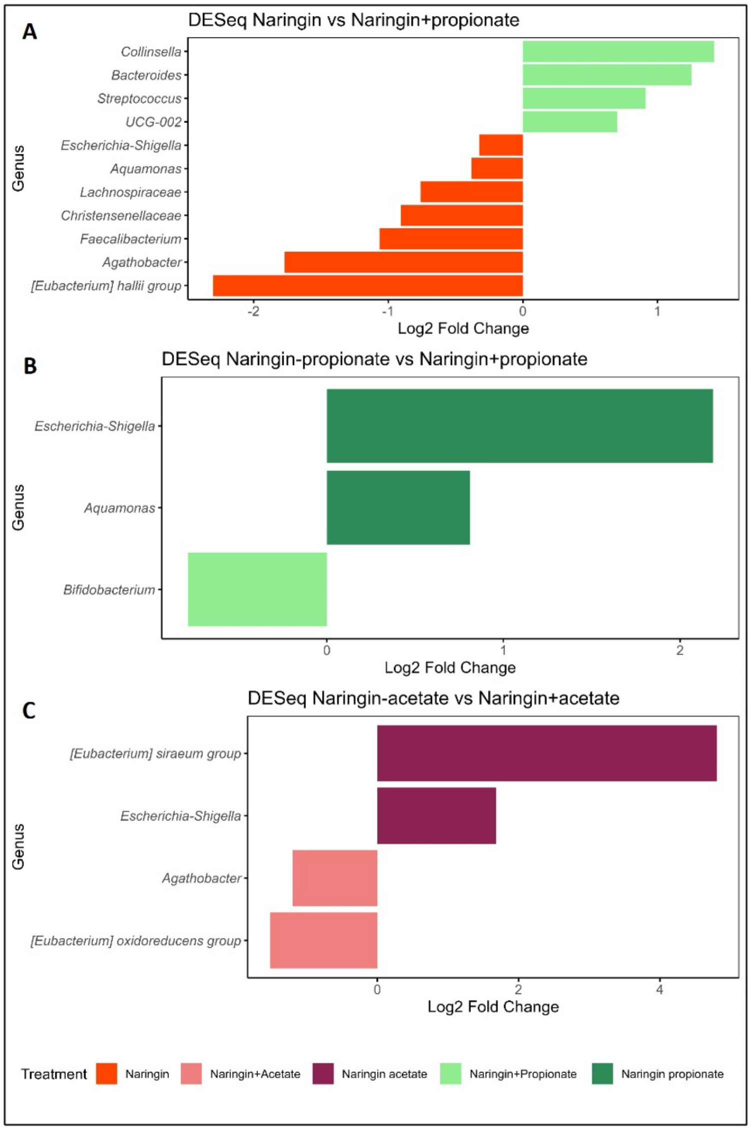

Figure S1. DESeq Analysis of GM: Treatment comparisons. Panel A presents the significant GM comparison in Donor A, while panels B and C illustrate the significant GM comparisons in Donor B at 24h of fecal fermentation. Panel A: comparison between Naringin *versus* Naringin+Propionate in donor A; Panels B and C: comparisons between Naringin Propionate and Naringin+Propionate, and Naringin Acetate and Naringin+Acetate, in Donor B respectively.

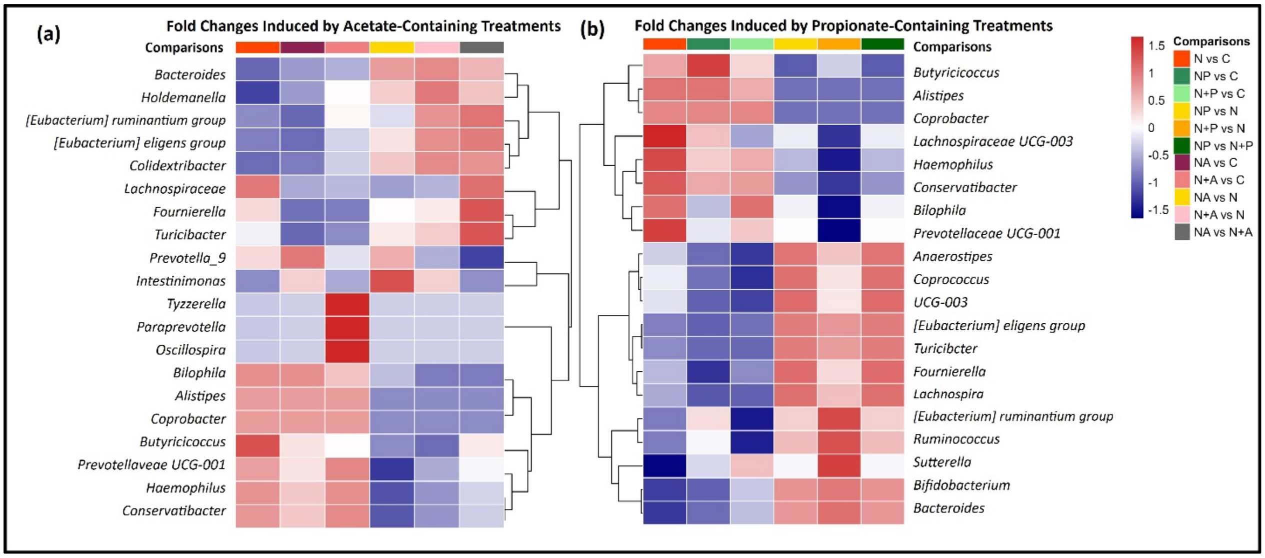

Figure S2. Heat map displaying DESeq-based comparisons of GM composition in response to Naringin treatments, highlighting tendencies in differential abundance across taxa in Donor A. Panel A shows the changes induced by acetate-containing treatments vs controls, while Panel B shows the changes induced by propionate-containing treatments vs controls. Treatment comparisons are labeled as follows: N vs C = Naringin *versus* Control, NP vs C= Naringin propionate *versus* Control, N+P vs C= Naringin+Propionate *versus* Control, NP vs N= Naringin propionate *versus* Naringin, N+P vs N= Naringin+Propionate *versus* Naringin, NP vs N+P= Naringin propionate *versus* Naringin+Propionate, NA vs C= Naringin acetate *versus* Control, N+A vs C= Naringin+Acetate *versus* Control, NA vs N= Naringin acetate *versus* Naringin, N+A vs N= Naringin+Acetate *versus* Naringin, NA vs N+A= Naringin acetate *versus* Naringin+Acetate. The heatmap displays Log Fold changes from DESeq analysis.

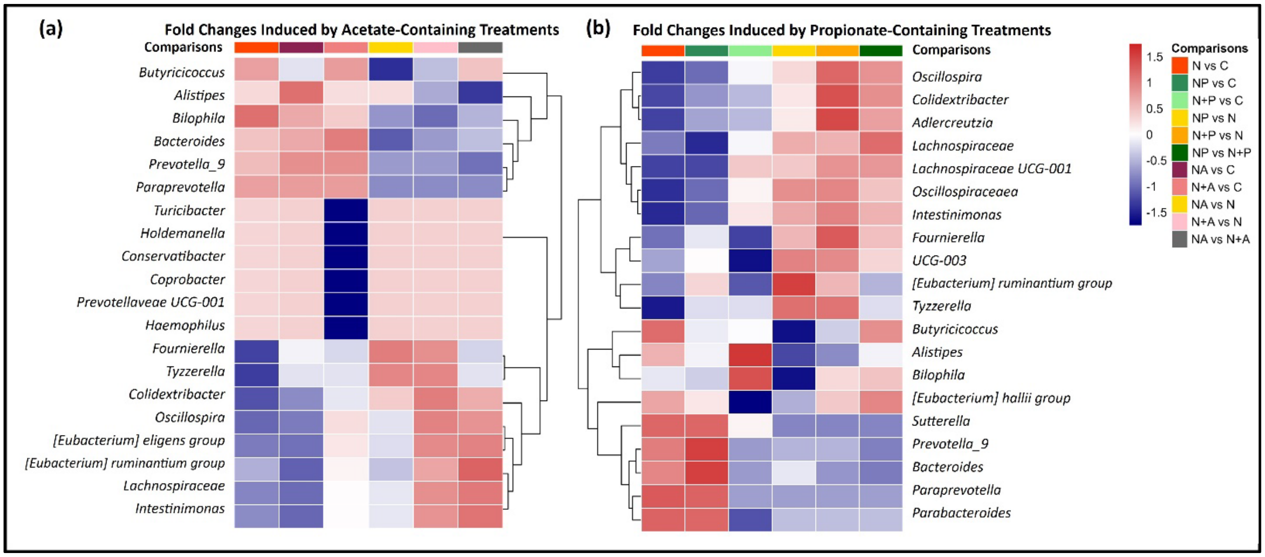

Figure S3. Overview of DESeq comparisons of GM from donor B in response to Naringin treatments in Donor B. Panel A shows the changes induced by acetate-containing treatments vs controls, while Panel B shows the changes induced by propionate-containing treatments vs

controls. Treatment comparisons are labeled as follows: N vs C = Naringin *versus* Control, NP vs C= Naringin propionate *versus* Control, N+P vs C= Naringin+Propionate *versus* Control, NP vs N= Naringin propionate *versus* Naringin, N+P vs N= Naringin+Propionate *versus* Naringin, NP vs N+P= Naringin propionate *versus* Naringin+Propionate, NA vs C= Naringin acetate *versus* Control, N+A vs C= Naringin+Acetate *versus* Control, NA vs N= Naringin acetate *versus* Naringin, N+A vs N= Naringin+Acetate *versus* Naringin, NA vs N+A= Naringin acetate *versus* Naringin+Acetate. The heatmap displays Log Fold changes from DESeq analysis.

Table S1. Anaerobic buffer composition.

**Reagents**	**g/100 mL**
K_2HPO_4 (Fisher)	0.88
KH_2PO_4 (Fisher)	0.68
$C_2H_3O_2SNa$ (Sigma)	0.1

Table S2. Nutritional SHIME® medium composition modified.

**Reagents**	**g/L**
Arabinogalactan (TCI)	1.20
Pectin (Acros Organics)	2.00
Xylan (TCI)	0.50
Glucose (Alfa Aesar)	0.40
Yeast extract (BioBasic)	3.00
Proteose peptone (Oxoid)	1.00
Mucin (Carl Roth by ProDigest)	2.00
L-cystein HCl (BioBasic)	0.50
Starch (Fisher)	4.00
*KH_2PO_4 (Fisher)	4.76
*Na_2HPO_4 (Fisher)	2.66

*The addition of KH_2PO_4 and Na_2HPO_4 constitutes the buffer Sørensen to stabilize the medium pH to avoid a drastic drop during the fecal fermentation.

Following the 45-minute residence period after nutritional medium delivery, 60 mL of pancreatic and bile juices were introduced, allowing for a 1 hour and 30-minute residence time at a pH of 6.8 to replicate the transition into the small intestine. The composition of the pancreatic juice is provided in the table below.

Table S3. Pancreatic juice composition

**Reagents**	**g/L**
$NaHCO_3$	12.5
Bovine Bile salts (Difco 212820)	6.0
Pancreatin 4Xusp	0.9

Following this, pre-digested contents were aliquoted in duplicated for each treatment (10mL) in 15mL tubes and immediately frozen and stored at -80°C until the fecal fermentation evaluation.

Sampling schedule

Fecal fermentation sampling **Donor _______ Date: __________**

- DNA= 2x500 μL. Centrifuge 8 min max speed, 4°C à pellet stored at -20°C
- SCFA= supernatant from DNA centrifuge to aliquot 1x125 μL + 1x375 μL stored at -80°C/ collect 400 μL and split 125 μL and 275 μL.

TIME	0	1	2	4	8	24
TREATMENT	DNA – SCFA	SCFA				DNA – SCFA
Control A						
Control B						
Naringin A						
Naringin B						
NAA A						
NAA B						
N+A A						
N+A B						
NPA A						
NPA B						
N+P A						
N+P B						

Control: no treatment
Naringin: Naringin
NAA: Naringin-acetate
N+A: Naringin + Acetate
NPA: Naringin-propionate
N+P: Naringin + Propionate
A: replicate A
B: replicate B

Table S4. Characterization of the donors

**Donor**	**Age**	**Sex**	**Nationality**	**Diet**
1	28	F	Italian	Pescetarian diet
2	33	M	Mexican	Flexitarian diet
